# Laterality of Attentional Networks in Patients With Cerebral Small Vessel Disease

**DOI:** 10.3389/fnagi.2020.00021

**Published:** 2020-03-20

**Authors:** Shanshan Cao, Jun Zhang, Zhiqi Wang, Wen Pan, Yanghua Tian, Panpan Hu, Qiang Wei, Jingye Wang, Xiuli Shi, Kai Wang

**Affiliations:** ^1^Department of Neurology, The First Affiliated Hospital of Anhui Medical University, Hefei, China; ^2^Anhui Province Key Laboratory of Cognition and Neuropsychiatric Disorders, Hefei, China; ^3^Department of Neurology, The Second Affiliated Hospital of Anhui Medical University, Hefei, China; ^4^Collaborative Innovation Center of Neuropsychiatric Disorders and Mental Health, Hefei, China

**Keywords:** cerebral small vessel disease, cognition, attention function, cognitive impairment, lateralization

## Abstract

**Introduction:** Problems with attention are common in patients with cerebral small vessel disease (CSVD). The normal human brain exhibits functional and structural asymmetry. However, it is unknown whether there is lateralization of attention in patients with CSVD.

**Objective:** This study aims to investigate attention separately in both cerebral hemispheres in patients with CSVD using the computer-based Lateralized Attention Network Test—Revised (LANT-R).

**Methods:** The total number of subjects included was 58, which includes the CSVD (*N* = 35) and healthy control (HC, *N* = 23) groups. All subjects completed the LANT-R paradigm and neuropsychological background tests.

**Results:** The results indicate that there is an left hemisphere (LH) lateralization in orienting network efficiency in the HC group. However, this lateralization was not apparent in the CSVD group. Furthermore, the difference between groups was significant (interaction *P* = 0.02). In addition, the scores of subjects in the CSVD group are lower in several cognitive domains, including attention function, memory function, information processing speed, and executive function, compared with the controls.

**Conclusion:** Patients with CSVD change in the lateralization of attention compared with the normal elderly. The decrease in attention in patients with CSVD might be caused by the reduced ability of selecting useful information in the LH.

## Introduction

Cerebral small vessel disease (CSVD) refers to the clinical, cognitive, imaging, and pathological manifestations of cerebrovascular syndrome caused by small vessel arteries, arterioles, capillary, and venule change (Chen et al., 2019). The major imaging features of CSVD mainly include white matter hyperintensities (WMHs), enlarged perivascular space (EPVS), cerebral microbleeds (CMBs), and lacunar infarcts (LI; French et al., 2014). The clinical manifestations of CSVD include a wide range of symptoms, which are as follows: typical stroke symptoms, varying degrees of cognitive impairment from mild impairment to dementia (Meissner, 2016). Some studies have shown that CSVD is an important cause of ischemic and hemorrhagic stroke, dementia, and depression (Pantoni, 2010). About 20–30% of patients experience ischemic stroke caused by CSVD (Rockwood et al., 2000). With the development of magnetic resonance imaging (MRI) technology, increasing numbers of patients with CSVD have been clinically diagnosed, and the prevalence of CSVD is about 6 to 10 times that of large blood vessel stroke (Longstreth et al., 2002; Black, 2011).

CSVD is very common in the elderly, WMHs occur in approximately 80% of Caucasians aged over 60 years (de Leeuw et al., 2001). In a healthy elderly population without cognitive impairment, the CMBs attack rate is 11–25% (Poels et al., 2011). With the acceleration of the aging process of the population, the incidence of cerebral vascular diseases is also on the rise. Among many risk factors for CSVD, age and hypertension are the clearest risk factors. The incidence of WMHs increases with age, with an average age of 10 years and an increase of two to three times (Turk et al., 2015). Meanwhile, studies have found that the detection rate of LI at 60 years old is 6% to 7%, while the detection rate at 80 years old is increased to 28% (Vermeer et al., 2007).

Recent data indicate that up to 5% of people over the age of 65 have varying degrees of vascular cognitive impairment (VCI; Rockwood et al., 2000). CSVD is an important cause of VCI in the population (Pantoni, 2010), in addition to increasing the risk for Alzheimer’s disease and dementia (Snowdon et al., 1997; Barnes et al., 2013). It has been estimated that there will be 135.5 million people with dementia worldwide by 2050. In 2010, $640 billion was spent globally to care for people with dementia, and this is expected to reach $1 trillion by 2030. These huge costs have imposed a heavy burden on the society, economy, and family life of patients (Robinson et al., 2015).

Previous studies have suggested that CSVD may reduce cognitive function (Pinter et al., 2015) and patients’ quality of life. Attention is an important component of cognitive function and a starting process for many cognitive processes. Attention helps us respond to external things faster and more accurately, and it also helps us select useful information and ignore useless information (Chica et al., 2012). Attention is the ability to deploy the resources of the brain to optimize performance toward behavioral goals (Atkinson and Braddick, 2012). This is an indispensable psychological process from which all psychological processes are generated and implemented. It is also the process by which the brain allocates appropriate resources for related sensory stimulation processes.

In 1990, Posner and Peterson proposed the “attention network theory” according to anatomical structure and functional MRI (fMRI) evidence, which divided the attention network into three independent components comprised of the alerting network, orienting network, and executive control network (Posner and Petersen, 1990; Petersen and Posner, 2012). The alerting network is the means by which the body maintains a sensitive functional state to accept input information. It mainly relies on the activation of the parietal and frontal lobes of the right hemisphere (RH), and is related to the function of the subcortical noradrenergic system (Coull et al., 2001; Fan et al., 2003). The orienting network is the means by which the brain selects useful information from all input information by shifting attention from one area or one object to another. It is mainly related to the function of the superior parietal lobe (Andersen et al., 1997), and some fMRI-based studies have suggested that the parietal, frontal, and temporoparietal junction of the RH also participates in the orienting network (Corbetta et al., 2000; Corbetta and Shulman, 2002). Studies have shown that blocking cholinergic input may affect the ability to shift attention (Davidson and Marrocco, 2000). The executive control network refers to the means by which the brain resolves conflict. The anterior cingulate cortex and the anterior frontal lobe participate in this network (Bush et al., 2000), and the dopamine system is functionally related (MacDonald et al., 2000). In 2002, Posner and Fan designed a simple computer-based paradigm—Attention Network Test (ANT; Fan et al., 2002) to measure the three network efficiencies of attention. Some studies that have applied ANT to patients with focal frontal and parietal lesions (Hu et al., 2013) and breast cancer (Chen et al., 2014) have confirmed that ANT is reliable.

Structural and functional cerebral asymmetry occurs during normal human brain development (Gunturkun and Ocklenburg, 2017). The asymmetry of the cerebral hemisphere extends almost all nervous systems in the human brain. At the structural level, it can be observed from the regional-specific expression asymmetry of certain genes (Karlebach and Francks, 2015). The posterior occipital lobe and temporal lobe of the left hemisphere (LH) are wider than in the RH, while the prefrontal lobe of the RH is wider than in the LH (Geschwind and Galaburda, 1985). These differences also yield an advantage to one cerebral hemisphere in many cognitive functions, especially attention function (Brooks et al., 2014). Kinsbourne’s theory assumes that there are attentional gradient differences within and across the visual fields (Làdavas et al., 1990). Another common model of spatial attention is called hemispatial theory (Nobre et al., 1997). The LH only controls attention toward the right visual field, whereas the RH is capable of controlling attention toward both sides of the visual fields. This theory has also been confirmed by transcranial magnetic stimulation (Duecker et al., 2013; Duecker and Sack, 2015). Some studies also suggest that attention function is lateralized in two cerebral hemispheres (Mesulam, 1999; Brooks et al., 2014). As for the attention network, Xuan et al. (2016) has shown that the orienting function is only related to the red nucleus of the LH. However, some brain regions of the two brain hemispheres are activated in both alerting and executive functions (Xuan et al., 2016). The Lateralized Attention Network Test—Revised (LANT-R) paradigm, based on the ANT paradigm can assess the status of attention networks within each hemisphere.

The LANT-R is a modified version of ANT to measure the hemispherical differences in the efficiency of the attention network (Fan et al., 2009). It is worth mentioning that the LANT-R paradigm can examine the efficiency and interactions of the attentional networks separately in the RH and LH (Bourne, 2006). Greene’s study has proven that LANT is a reliable and sensitive method for testing the efficiencies of the three networks in the two cerebral hemispheres separately (Greene et al., 2008). Asanowicz et al. (2012) used the LANT paradigm to discover that alerting network did not differ across the hemispheres, with greater efficiency of the RH in the invalid orienting cue condition, and RH’s dominance in resolution of conflict. Bin Xuan proposed that the LH is more dominant in the efficiency of the orienting network under the valid cue condition. Through previous articles, we found that there may be hemisphere differences in the orienting network. However, most of the previous studies were based on younger populations, and we will conduct further research in the elderly population through LANT-R.

Some studies have proposed that patients with CSVD are more likely to have cognitive function decline, especially in attention, than normal people, as assessed using traditional neuropsychology paper tests, fMRI, and brain electrical methods (Hund-Georgiadis et al., 2001; Dey et al., 2016). However, these studies only focused on the global attention function of patients with CSVD and did not analyze the efficiencies of the three networks involved in attention further. If we want to explore the underlying mechanism of impaired attention in patients with CSVD, it is necessary to understand how the efficiencies of the three networks involved in attention changes and whether the brains of patients with CSVD exists lateralization. However, no study has yet examined the attention network in both cerebral hemispheres in patients with CSVD. According to previous studies, we know that attention function has a hemispherical lateralization in normal people. Because of the presence of lesions, we suspect that this balance of lateralization may be broken, leading to the disappearance of lateralization of attention in patients with CSVD.

## Materials and Methods

### Subjects

The features of CSVD in neuroimaging mainly include WMHs, LI, CMBs, and EPVS. The clinical symptoms of CSVD mainly include acute symptoms and subacute symptoms. The acute symptoms mainly include transient ischemic attack (TIA) and lacunar syndrome. The subacute clinical symptoms include cognitive impairment and dyskinesia (Román et al., 2002). As the onset of CSVD is often insidious, clinically heterogeneous, and typically has mild symptoms, it has been suggested that the selection of subjects with CSVD in clinical studies should be based on the more consistent brain imaging features (Erkinjuntti, 2002).

From July 2017 to July 2018, 35 patients with CSVD from outpatient and inpatient departments of the First Affiliated Hospital of Anhui Medical University, Hefei, China, were admitted to our study. The common symptoms of patients attending the clinic included dizziness, memory decline, and gait disorder. Some of the patients were identified from previous stroke and physical examinations. The inclusion criteria were as follows: (1) age between 50 and 80 years; (2) MRI suggesting the presence of LI (subcortical ≥1) with WMHs (Fazekas ≥2) or two or more other common imaging features of CSVD, including EPVS (basal ganglia ≥10); (3) the diameter of the LI lesion was 3–15 mm on T2 or fluid-attenuated inversion recovery phase; and (4) for patients with acute symptoms (including TIA, lacunar syndrome, etc.) and with a lesion on MRI, we required a maximum diameter of <20 mm (axial position) on diffusion-weighted imaging. Cognitive assessment of these patients was completed after 3 months from onset to reduce the impact of acute cerebral infarction.

The exclusion criteria were as follows: (1) intracranial and extracranial stenosis over 50%; (2) trial of ORG 10172 in Acute Stroke Treatment classification suggestive of cardiogenic stroke; (3) presence of cortical infarct or subcortical infarct, and a lesion diameter of >1.5 cm (non-acute); (4) non-CSVD-related WMHs (e.g., multiple sclerosis); (5) physical and mental disorders, alcohol addiction; (6) patients with dementia or tumors; (7) intracranial hemorrhage; (8) significant hearing or visually impaired persons, physical movement disorders that prevented cooperation during cognitive testing; (9) language barrier; and (10) MRI contraindications or known claustrophobia.

The participants in the healthy control (HC) group were relatives of patients with CSVD and social recruits studied in the same period who matched the demographic data of the patients with CSVD, including age, gender, and education level, and had no previous history of neurological diseases, mental illnesses, and the imaging showed no white matter high signal. The study was approved by the ethics committee of the First Affiliated Hospital of Anhui Medical University. All subjects provided written informed consent before the study.

### Magnetic Resonance Parameters

MRI scanning was completed at the University of Science and Technology of China using a 3.0T MRI scanner (Discovery MR750; GE Healthcare, Milwaukee, WI, USA).

A T1-weighted imaging sequence was used as follows: layer thickness = 5 mm, total surface area = 20 layers. A T2-weighted imaging sequence was used as follows: layer thickness = 5 mm, total laye*r* = 20 layers.

We did not include susceptibility weighted imaging sequence or T2-gradient-recalled echo sequences when performing MRI scans, so CMB lesions were not included in this study.

### Neuropsychological Test

The neuropsychological scale of the Chinese CSVD Clinical Evaluation Study was used to evaluate the global cognitive function and individual cognitive functions of all subjects. The scale refers to the National Institute of Neurological Disorders and Stroke and the Canadian Stroke Network (NINDS-CSN)-recommended cognitive assessment program for VCI (60-min version) and NINDS-CSN China Vascular Cognitive Impairment Assessment Program. We selected tests that are sensitive to CSVD-related cognitive impairment. The LANT-R paradigm was used to evaluate the attention of the subjects. The testers were all graduate or doctoral students in neurology who had passed the unified training.

### Overall Cognitive Function

We used the Montreal Cognitive Assessment scale (MoCA) to assess the overall cognitive function of all subjects. This scale consists of 30 items and was used to evaluate the subjects’ attention and concentration, executive functions, memory, language, visuoconstructional skills, conceptual thinking, calculations, and orientation. The total score ranges from 0 to 30, and the higher the total score, the higher the overall cognitive function level of the subjects (Nasreddine et al., 2005).

### Anxiety and Depression

Anxiety was assessed using the Generalized Anxiety Disorder-7 (GAD-7). The total score ranges from 0 to 21, and the higher the total score, the more severe the anxiety (Hinz et al., 2017). Depression symptoms were assessed using the Patient Health Questionnaire-9 (PHQ-9). The total score ranges from 0 to 27, and the higher the total score, the more severe the depression (Smarr and Keefer, 2011).

### Individual Cognitive Function

The Auditory Verbal Learning Test (AVLT) consists of three parts: immediate recall test, delayed recall test, and recognition test. First, the tester reads 15 words, and the subject recalls the 15 words immediately. The tester read the same 15 words five times, and each time the tester finished reading, the subject was asked to recall the words. The average of the five times the correct words were recalled was recorded as the subject’s “immediate memory test” score. About 30 min later, the subject was asked to recall the 15 words that the tester had read five times before. The correct number of words the subject could recall was recorded as the subject’s “delay memory test” score. Finally, the subject identified which words the tester had read before from 50 words, and the number of words correctly recognized were recorded as the subject’s “recognition test” score (Schoenberg et al., 2006).

The verbal fluency test (VFT) was used to evaluate the executive and language function of the subjects. This scale requires the subjects to name as many animals as possible within 1 min. The greater the number named, the better the subject’s executive and language function (Thurstone, 1957).

The digital span (DS) test consists of two parts, forward and backward, and was used to evaluate the attention of the subjects. This scale required the subjects to repeat the string number read by the tester in the forward and backward direction. The length of the sequence of numbers gradually increased. The score was recorded as the maximum sequence length of numbers that the subject could correctly repeat. The higher the score the subjects got, the better the participant’s attention (Yamamoto et al., 2011).

The Stroop Color Word Test (SCWT) was used to evaluate the subjects’ attention and executive function. This scale required the subjects to say the color of points, words, and color words as quickly and correctly as possible. The less time it took, the better the executive and attention function of the subjects (Stroop, 1935/1992).

The Trail Making Test (TMT) was also used to evaluate the subjects’ executive and attention function. This scale requires the subjects to link 25 numbers as quickly as possible in numerical order from 1 to 25. The shorter the time taken, the better the executive and attention function of the subjects (Selnes et al., 1991).

### The Lateralized Attention Network Test—Revised

At the beginning of the test, a fixed gaze point “+” appeared in the center of the computer screen. The subjects were required to gaze at the gaze point “+.” There was a rectangular frame on the left and right sides of the gaze point. After 150 ms, a cue would last for 100 ms. Then the subjects would see a set of five arrows appearing randomly in the left or right box in the vertical direction. The arrows appeared for 500 ms, and the subjects were required to judge the middle arrow’s (target arrow) direction as quickly as possible. If the target arrow pointed up, the left mouse button was pressed. If the target arrow pointed down, the right mouse button was pressed. Whether the direction of the target arrow was consistent with the direction of the other four arrows or not, there were two types of arrows: (1) consistent direction (five arrows point the same direction); (2) inconsistent direction (the target arrow’s direction is opposite to other four arrows). The subjects were required to respond within 1.70 s. The time from five arrows disappearing to the start of the next trial was approximately 2,000 ms to 12,000 ms, and the average time was 4,000 ms. The average time per trial was about 5,000 ms. The cues were divided into the following four types: (1) no cue: no box flashed before the arrows appeared; (2) double cue: before the arrows appeared, the two boxes flashed; (3) valid cue: one of the boxes flashed and the targets appeared in the flashing box; and (4) invalid cue: one of the boxes flashed and the targets appeared in the non-flashing box.

The whole test consisted of four rounds, and each round took approximately 7 min. The total time taken was approximately 30 min. The paradigm automatically recorded whether the response was correct or not and the reaction time (RT) under the correct response.

**Figure 1:**
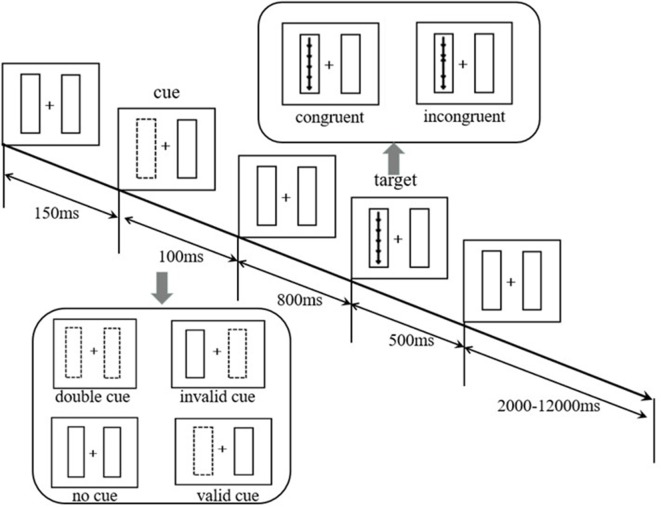
Schematic of Lateralized Attention Network Test—Revised (LANT-R).

Each subject performed 24 practice trials before the official test. During the practice period, the subjects received feedback on whether they had responded correctly and their RT under the correct response after each trial (no feedback on official trials). Figure 1 illustrates the paradigm flow.

### The Calculation of the Efficiencies of the Three Attention Networks

(1) Alerting network efficiency = RT no cue − RT double cue
(2) The process of selecting information consisted of three basic components: disengaging attention from its current focus, moving attention to a new target or modality, and engaging attention at a new target. Disengaging attention can be derived by comparing the RT of the invalid cue and double cue; a deficit in the moving of attention can be inferred when the RT to targets is slow, regardless of where attention was engaged prior to target appearance; a deficit in attention engagement can be indexed if there is an RT deficit despite the targets having been validly cued and the cue-to-target interval being long enough to allow attention to move to the new target.
Orienting network efficiency under valid cue condition (Orienting) = Moving + Engaging = RT double cue − RT valid cue
Orienting network efficiency under invalid cue condition (Reorienting/Disengaging) = RT invalid cue − RT double cue
Orienting network efficiency (Validity effect) = Disengaging + (Moving + Engaging) = RT invalid cue − RT valid cue
(3) Executive control network efficiency = RT inconsistent − RT consistent (Fan et al., 2009).

RT above or below 2 standard deviations (4.31%) were considered as outliers and then disregarded from analyses (Spagna et al., 2014). Greene et al. (2008) has confirmed that the LANT is a useful extension of the ANT, yielding significant estimates of the attentional networks in each hemisphere. We have a fixed “+” as the center. The arrow on the left side of “+” was regarded as the information on the left side of the field of view. The arrow on the “+” side was regarded as the information on the right side of the field of view. An image of one side of the field will transmit the contralateral hemisphere. Therefore, the three network efficiencies of the cerebral hemispheres based on the RT of the subjects under different conditions in the contralateral field of view were calculated.

### Statistical Analysis

The demographic data for the CSVD and HC groups were compared using a Chi-square test or Student’s *t*-test. Intra-group differences on the neuropsychological tests and for the efficiencies of the three networks in the LH and RH were compared using the Student’s *t*-test. The efficiencies of the three networks in the LH and RH were analyzed using a repeated measures analysis of variance between the CSVD and HC groups. We also calculated the efficiencies of the three global networks from the average values in the LH and RH. A Pearson’s correlation analysis was used to assess the relationship between the efficiencies of the three global networks for the total average RTs as well as for other neuropsychological tests. Differences with a two-tailed *P* < 0.05 were considered statistically significant.

## Result

### Demographic and Neuropsychological Test Data

The demographic data, global cognitive function, and individual cognitive function results of the CSVD and HC groups are shown in Table 1. There was no significant difference between the two groups in terms of age, education years, gender, anxiety, and depression. However, the performance of the CSVD group was significantly worse than the HC group with regard to global cognitive function (i.e., MoCA), attention function (i.e., DS-backward test, DS-forward test), memory function (i.e., the Immediate Recall and Delayed Recall test), information processing speed (i.e., TMT-A), executive function (i.e., TMT-B), and prefrontal lobe function (i.e., VFT-animal; *P* < 0.05).

**Table 1:** Demographic data and neuropsychological background tests between the CSVD and HC groups.

	CSVD group (*n* = 35) Mean ± SD	Healthy control (*n* = 23) Mean ± SD	*t*/χ^2^	*P*	Cohen’s *d*/ϕ
Age (years)	62.49 ± 8.80	59.96 ± 7.79	1.14	0.26	0.31
Male	23	12	1.06	0.32	0.14
Education (years)	8.80 ± 3.15	10.22 ± 3.42	−1.63	0.11	−0.44
PHQ-9	5.06 ± 4.16	3.90 ± 3.37	1.02	0.31	0.28
GAD-7	2.61 ± 3.58	2.81 ± 3.17	−0.18	0.86	−0.05
**Global cognition**
MoCA	22.46 ± 3.19	25.04 ± 2.26	−3.49	<0.001	−0.94
**Attention/concentration function**
WAIS Digit Span (forward)	6.91 ± 1.36	8.05 ± 1.33	−3.08	<0.05	−0.84
WAIS Digit Span (backward)	3.88 ± 1.17	5.05 ± 1.73	−2.91	<0.05	−0.80
**Memory (AVLT) function**				
Immediate recall	6.82 ± 1.60	8.92 ± 1.58	−5.34	<0.001	−1.46
Delayed recall	6.29 ± 2.86	9.61 ± 2.79	−4.40	<0.001	−1.18
Recognition	12.91 ± 1.87	13.57 ± 1.24	−1.51	0.14	−0.41
**Executive function**				
Trail Making Test B (s)	168.80 ± 71.96	116.14 ± 53.96	2.715	<0.05	0.74
Stroop Word Test (s)	26.07 ± 8.94	22.26 ± 6.15	1.46	0.15	0.39
Stroop Interference Test (s)	41.28 ± 17.70	34.74 ± 10.59	1.48	0.14	0.40
**Information processing**				
Trail Making Test A (s)	86.02 ± 33.24	55.13 ± 18.80	3.38	<0.01	0.92
**Language function**				
Verbal fluency (animal)	14.97 ± 4.94	17.74 ± 4.41	−2.57	0.01	−0.70

### Efficiencies of the Three Networks

Table 2 lists the average RTs under different conditions in the CSVD and HC groups.

**Table 2:** Average reaction time (RTs) under different conditions in the CSVD and HC groups.

Group	*n*	Flanker type	Hemisphere	No cue Mean RT ± SD	Valid cue Mean RT ± SD	Invalid cue Mean RT ± SD	Double cue Mean RT ± SD
CSVD group	35	Congruent	LH	1,007.30 ± 186.63	913.77 ± 167.06	1,018.60 ± 177.37	971.70 ± 169.46
			RH	983.15 ± 151.66	922.39 ± 164.57	1,002.90 ± 172.02	1,001.40 ± 191.60
		Incongruent	LH	1,084.50 ± 167.48	1,031.30 ± 162.17	1,152.20 ± 169.59	1,084.50 ± 167.48
			RH	1,082.40 ± 156.72	1,022.50 ± 160.11	1,141.50 ± 152.13	1,082.40 ± 156.72
HC group	23	Congruent	LH	933.78 ± 156.46	838.06 ± 155.27	933.83 ± 150.21	933.78 ± 156.46
			RH	899.76 ± 161.39	868.75 ± 164.51	921.71 ± 159.94	899.76 ± 161.39
		Incongruent	LH	1,047.30 ± 157.58	970.06 ± 180.11	1,090.60 ± 140.15	1,047.30 ± 157.58
			RH	1,024.10 ± 138.59	959.09 ± 163.39	1,075.90 ± 143.05	1,024.10 ± 138.59

### Alerting Network Efficiency

**Figure 2:**
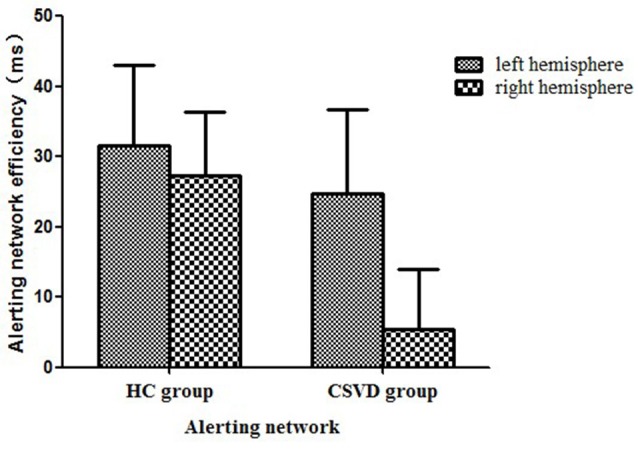
Alerting network efficiency in healthy control (HC) and cerebral small vessel disease (CSVD) groups.

We calculated the alerting network efficiency and found that there was no advantage between hemispheres in the CSVD group (LH vs. RH, 24.78 ± 70.49 vs. 5.48 ± 50.43, *t* = 1.35, *P* = 0.19, Cohen’s *d* = 0.23) or HC group (LH vs. RH, 31.62 ± 54.47 vs. 27.33 ± 43.40, *t* = 0.30, *P* = 0.77, Cohen’s *d* = 0.06), as shown in Figure 2.

### Orienting Network Efficiency

We analyzed differences in orientation function under different cue conditions. We found no difference between hemispheres in either the CSVD group (LH vs. RH, 62.33 ± 63.24 vs. 43.64 ± 55.03, *t* = 1.15, *P* = 0.26, Cohen’s *d* = 0.20) or the HC group (LH vs. RH, 53.34 ± 52.49 vs. 64.17 ± 41.66, *t* = −0.78, *P* = 0.44, Cohen’s *d* = −0.16) in terms of orienting function under invalid cue conditions.

The HC group had an LH advantage in orienting (LH vs. RH, 54.84 ± 33.84 vs. 20.70 ± 34.64, *t* = 3.80, *P* = 0.001, Cohen’s *d* = 0.79) under valid cue conditions, but the advantage of the LH disappeared in the CSVD group (LH vs. RH, 50.98 ± 52.63 vs. 54.03 ± 40.12, *t* = −0.28, *P* = 0.79, Cohen’s *d* = 0.05).

**Figure 3:**
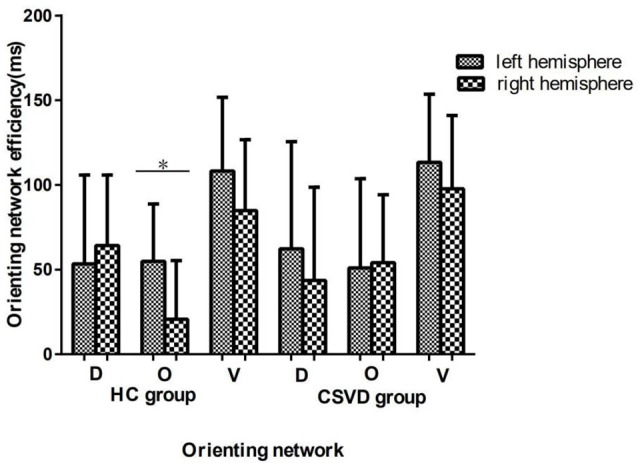
Orienting network efficiencies in HC and CSVD groups. *Significant at the 0.05 level (2-tailed).

As for the validity effect, we did not find a significant difference in the cerebral hemispheres in either the HC group (LH vs. RH, 108.18 ± 43.51 vs. 84.87 ± 41.77, *t* = 2.04, *P* = 0.05, Cohen’s *d* = 0.43) or the CSVD group (LH vs. RH, 113.31 ± 40.29 vs. 97.67 ± 43.35, *t* = 1.65, *P* = 0.11, Cohen’s *d* = 0.28), as shown in Figure 3.

### Executive Control Network Efficiency

**Figure 4:**
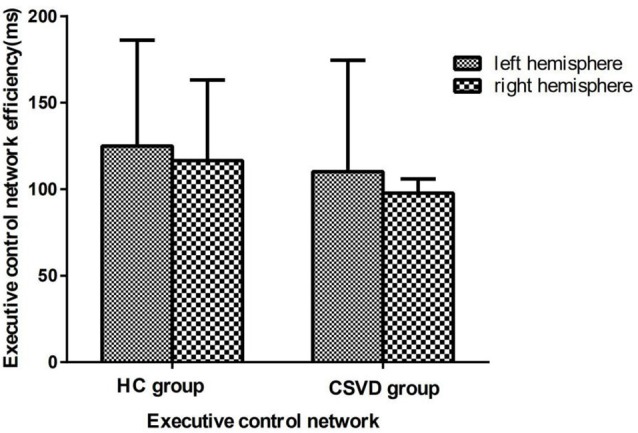
Executive control network efficiency in HC and CSVD groups.

We calculated the executive control network efficiency and found that neither the CSVD group (LH vs. RH, 110.19 ± 64.33 vs. 97.71 ± 48.91, *t* = 1.23, *P* = 0.23, Cohen’s *d* = 0.21) nor the HC group (LH vs. RH, 125.00 ± 61.11 vs. 116.50 ± 46.60, *t* = 1.04, *P* = 0.31, Cohen’s *d* = −0.42) had a hemispheric advantage. Figure 4 shows the executive network efficiency in the CSVD and HC groups.

### Interaction Effect

**Figure 5:**
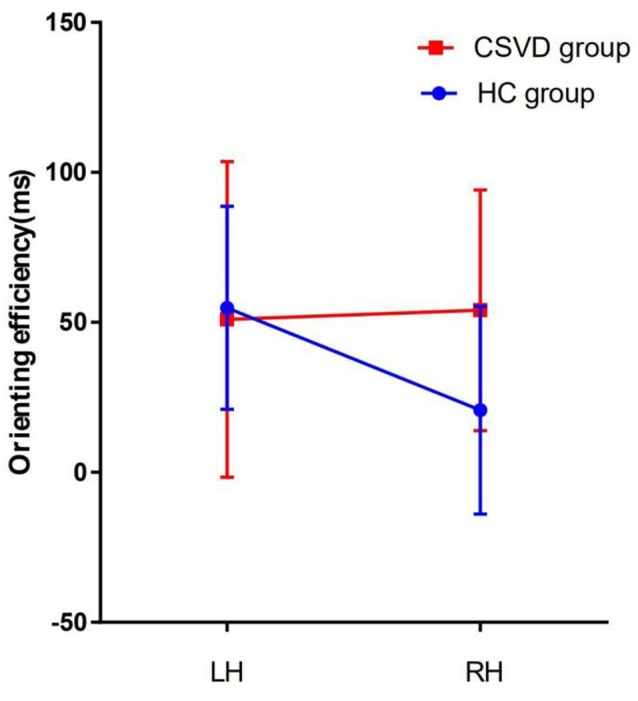
Interaction effect between HC group and CSVD group in orienting efficiency.

After further comparison between groups, an interaction analysis [2 (HC, CSVD) × 2 (LH, RH); *F* = 5.76, interaction *P* = 0.02, *η*^2^ = 0.09] showed significant differences between groups in orienting efficiency, as shown in Figure 5.

### Intergroup Analysis of the Ipsilateral Cerebral Hemisphere

**Figure 6:**
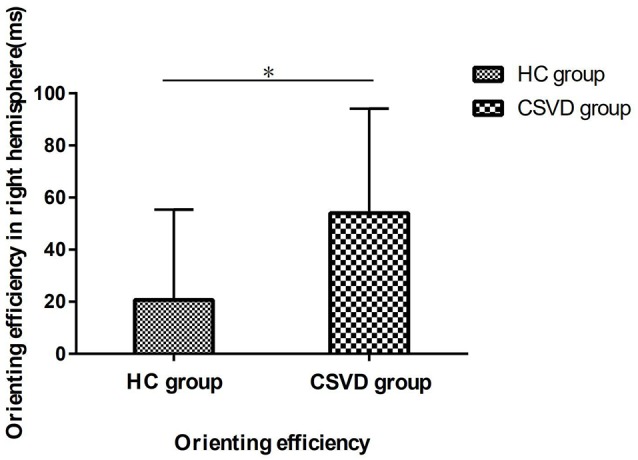
The orienting efficiency in the right hemisphere (RH) in HC and CSVD group. *Significant at the 0.05 level (2-tailed).

Based on the above results, we performed an analysis of the orienting efficiency of the ipsilateral hemispheres in order to find out which side of the hemisphere changes the orienting pattern. We found that the difference in orienting efficiency mainly exists in the RH between the two groups (HC vs. CSVD, 20.70 ± 34.64 vs. 54.03 ± 40.12, *t* = 3.26, *P* = 0.002, Cohen’s *d* = −0.88), as shown in Figure 6.

### Correlations

**Figure 7:**
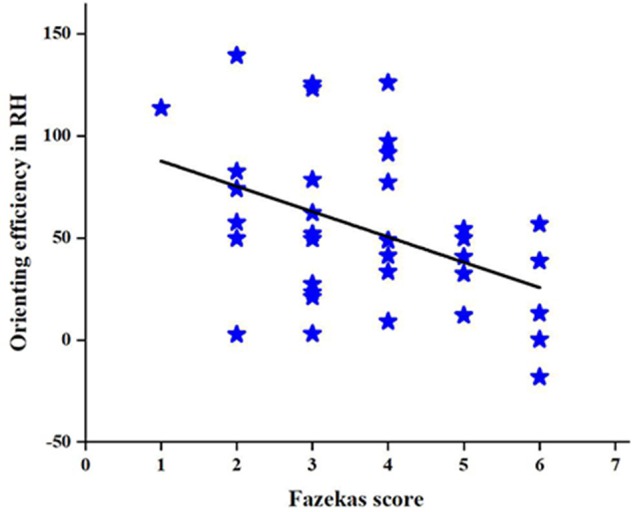
Correlation between the Fazekas score and orienting efficiency in patients with CSVD.

To further investigate the relationship between the change in the orienting efficiency of the RH and CSVD characteristic indicators in patients with CSVD, we performed a correlation analysis to find a correlation between the Fazekas score and orienting efficiency in patients with CSVD (*r* = −0.43, *P* = 0.01), as shown in Figure 7. However, no correlation is found between age and orienting efficiency in RH.

**Figure 8:**
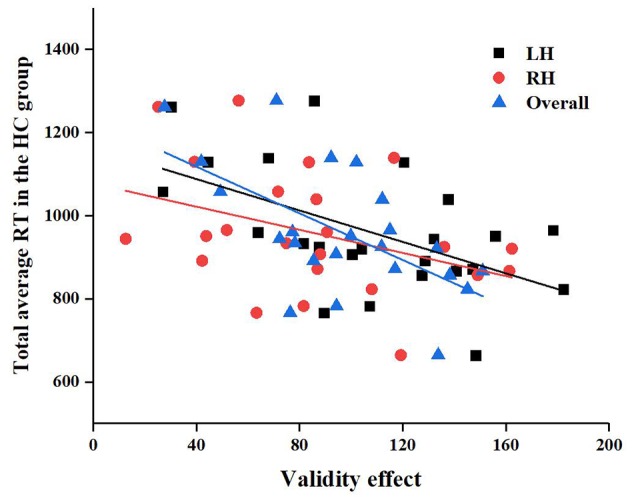
Correlation between left hemisphere (LH), RH, and overall validity effect and total average reaction time (RT) in the HC group.

In addition, we also found that in the HC group, the LH (*r* = −0.51, *P* = 0.01), RH (*r* = −0.43, *P* = 0.04), and total brain (*r* = −0.58, *P* = 0.004) validity effects were significantly correlated with the total mean RT, as shown in Figure 8, but there were no significant correlations in the CSVD group, as shown in Figure 8.

## Discussion

There is evidence to suggest that patients with CSVD exhibit cognitive impairment. In this study, we first used the sensitive and objective LANT-R paradigm to test the attention function in the two cerebral hemispheres of the subjects. Our data suggest that the HC group has an LH advantage in orienting, and this does not appear in the CSVD group. Specifically, this change in orienting function is caused by a difference in the orienting function of the RH. We also find a correlation between the Fazekas score and orienting efficiency in patients with CSVD in RH. In addition, the data suggest that the global cognitive function of patients with CSVD are worse than normal elderly controls, especially in terms of attention function, memory function, executive function, and information processing speed.

Our data suggest that the HC group has LH advantage in selecting information, and this is consistent with previous research results. Heilman proposed early that the RH participates in the information processing of the bilateral view field, while the LH only receives information from the right field (Glick et al., 1982). Some studies have also expressed the same view. Through the use of positron emission tomography technology, Corbetta et al. (1993) found that both sides of the top lobe were active when paying attention to the right view side, while when people’s attention shifted to the left side of the field, only the superior parietal lobe was active. Bin Xuan proposed that the orienting network efficiency under a valid cue condition was only associated with the left red nucleus (Xuan et al., 2016).

We all know the famous HAROLD model, and Cabeza believes that hemisphere asymmetry in older people weakens when performing some tasks compared with that in young people (Cabeza, 2002). They believe that the weakening of the asymmetry of the brain in the elderly is to compensate for the cognitive decline caused by aging. As for patients with CSVD, the presence of lesions is an injury to them. We guess that these lesions make patients with CSVD make some compensation in order to compensate the orienting function. Based on our results, we further speculate that this compensation may come mainly from the RH. In addition, orienting function is closely related to the cholinergic system. The presence of lesions in the patients with CSVD may affect the synthesis and release of acetylcholine, which may alter the hemisphere asymmetry of orienting function. However, this was only our data-based guess and needs to be further confirmed by fMRI or other research methods that reflect brain function.

As for disengaging function under invalid cue conditions, there is no advantage hemisphere in the HC group. This phenomenon is consistent with the bi-directional gradient theory proposed by Handy et al. (1996). There are inhibitory effects and activation effects in orienting function. The activation effect always exists regardless of whether the cue is valid, and the inhibition effect is activated when the cue is invalid. The inhibitory effect on the right field of view is greater than the inhibitory effect on the left field of view. In the case of uncertainties about time and space, it is less likely for the subject to focus all of their attention on the induced position (where the cue occurs), thereby reducing the asymmetry under invalid cue conditions (Rhodes and Robertson, 2002).

We found that the LH have advantage over the RH for the orienting function in the HC group under all cue conditions, and that the difference between the hemispheres was 24 ms, which trended toward significance. This phenomenon may be caused by our small sample size. We did not find lateralization of the validity effect in patients with CSVD. The validity effect is a combination of disengaging and orienting under all cue conditions. This suggests that there is a difference between the ability of the patients with CSVD to select useful information and that of normal elderly controls.

However, the above results contrast with the results of Rhodes and Robertson (2002), which suggest that there is no field of view advantage under invalid cue conditions. This inconsistency may be due to the fact that Rhodes’ experiment used young people, but our study was conducted in elderly subjects. Meanwhile, some other studies have suggested that the RH has advantages in orienting function or attention function (Corbetta et al., 2000; Corbetta and Shulman, 2002; Erel et al., 2019). We believe that the reason for these differences may be that previous studies focused more on the entire attention function or orienting function without further detailed analysis based on the attention network or conditions of the cues.

The results of our study suggest that there is no lateralization of alerting in normal elderly controls. At present, there is no consensus about whether lateralization exists in the alerting network. A neuroimaging-based study suggested that the RH and the thalamus play major roles in the alerting network (Ross and Monnot, 2008), while other studies have suggested that alerting networks are primarily associated with the LH (LeMay, 1976; Sturm and Willmes, 2001). In addition, according to recent literature, the alerting network is mostly associated with the thalamus, and the frontal and parietal cortex. There is also no consensus about whether lateralization exists in the executive control network. Previous studies have suggested that the RH, including regions such as the right prefrontal lobe and the right anterior cingulate gyrus, shows advantage for resolving conflicts and reaction inhibition (Aron et al., 2003; Hampshire et al., 2010). Several studies have shown that the midline frontal and prefrontal cortex are activated during execution.

Meanwhile, we find strong correlation between the Fazekas score and the orienting efficiency in RH in patients with CSVD, the orienting function, and the total average RT between patients with CSVD and healthy elderly. The presence of WMHs signifies traumas to the brain like the role of age in the HAROLD model. In order to adapt to the damage caused by the WMHs, the brain will perform an adaptive compensation mechanism. However, as the intensity of the trauma increases, the brain’s ability to compensate weakens. However, the correlation between age and LANT-R is not significant. Therefore, we suspect that the influence of the severity of WMHs on cognition is more important in patients with CSVD. The process of selecting information in normal elderly controls had a significant effect on attention, but there was no association between the process of selecting information and attention in patients with CSVD. We suspect that the reduced attention of patients with CSVD may be due to difficulties in selecting information.

Similar studies have suggested that the emotional state may affect the neuropsychological assessment results of subjects, so we used the GAD-7 and PHQ-9 scales to evaluate the emotional state of subjects. There were no significant differences between the two groups with regard to anxiety and depression scores, which reduces the possible influence of emotional state on our conclusion.

Our study has two limitations. First, our study was a cross-sectional study. Thus, the above conclusion can only be based on the existing data, but these data cannot clarify the specific reasons for the disappearance of the hemispheric advantage in patients with CSVD under the valid cue condition. In future studies, we will conduct fMRI analyses to determine the relevant neural mechanisms underlying this. Second, CSVD imaging markers include LI, WMHs, CMBs, and perivascular space enlargement; however, the patients enrolled in our study had mainly WMHs and LI on MRI, and thus we could not classify the lesion type and location.

## Conclusion

Our study represents a step toward understanding attention deficits in patients with CSVD. Our findings revealed that the patients with CSVD exhibited different patterns compared with the normal elderly. At the same time, we find that patients with CSVD are seriously damaged in several cognitive functions.

## Data Availability Statement

All datasets generated for this study are included in the article.

## Ethics Statement

The studies involving human subjects were reviewed and approved by the Ethics Committee of the First Affiliated Hospital of Anhui Medical University. The patients/subjects provided their written informed consent to participate in this study.

## Author Contributions

SC: performed the analysis and wrote the manuscript. KW: substantial contribution to the conception of the work. KW, YT, JZ, and SC: design of the work. SC, JZ, ZW, WP, PH, QW, JW, and XS: acquisition and analysis. SC, ZW, and WP: interpretation of data for the work. SC: drafting the work. KW and JZ: revising it critically for important intellectual content. SC, JZ, ZW, WP, YT, PH, QW, JW, XS, and KW: final approval of the version to be published and agreement to be accountable for all aspects of the work ensuring the questions related to the accuracy or integrity of any part of the work are appropriately investigated and resolved by all authors.

## Conflict of Interest

The authors declare that the research was conducted in the absence of any commercial or financial relationships that could be construed as a potential conflict of interest.
